# Much Bothering about a Non-Essential for Success in Practice

**Published:** 1901-02

**Authors:** B. F. Arrington

**Affiliations:** Goldsboro, N. C.


### MUCH BOTHERING ABOUT A NON-ESSENTIAL FOR SUCCESS IN PRACTICE.

BY DR. B. F. ARRINGTON, GOLDSBORO, N. C.

In the *Items of Interest* for October, 1900, page 751, we see it stated: "A question that is unsolved and is bothering not only the dentists of America, but also of England, and no doubt all of the thinking men of the profession in the world is, 'Why teeth decay?'"

As regards practical benefits to be derived, we might as well be bothering about and asking why eyesight fails, or muscle and sinew weaken, what is the cause of cancer, and why people die short of advanced age? Such querying would be about as reasonable and would possibly develop as satisfactory results for evidence of cause.

To know the cause, if possible, of any disease or abnormal condition of the human frame, whether of the osseous structure or soft tissues, is desirable, and must be anticipated as a possible ultimate result of earnest effort in scientific investigations. But will it not be well for us, in our efforts for more light and knowledge of causes, to consider and accept the fact that possibly there are freaks and features in the physical make-up of man that may never be so definitely comprehended and so explained as to profit either the afflicted or those who administer for relief.

Caries of teeth is a feature of physical defect that may present at any period of life from early childhood to old age, and has thus prevailed, in all probability, with some peoples since the earliest records of the human family (evidence recorded justifies such belief), and may continue to the end of time.

One fact connected with caries and worthy of thought is, that caries in attacking teeth, like cancer fleshy tissue, has special preference for certain parts, but varies and sometimes attacks where least expected and where there is no recognition of cause or evidence to justify belief that animate cause does exist in accord with the popular modern idea (germ theory); not even with the aid of best magnifying glasses are signs of cause detectable. It is a well-known fact that decay often presents on the buccal and lingual surfaces of molars (smooth surfaces) that are carefully cleansed and kept free from deposits and environments favorable for germ production and lodgement for development and action.

Many questions in medicine pertaining to cause of disease, as this "question" of caries in dentistry, are unsolved, but treatment goes on and cures are effected, and so it must be in dentistry. To be able to recognize and name correctly a disease when first seen is more important for successful treatment than to know the cause. It is well to know cause if possible, but if known definitely it is no guarantee against the existence of disease or of successful treatment.

A reasonable exercise of mind with dentists about the causes of caries, and more earnest effort to learn how to check the ravages of the disease and preserve the teeth in a normal state as near as possible would be more in range of possibility and in keeping with professional duty. Scientists will carry on the work of investigation and discovery of causes, and furnish facts concerning caries which dentists, as a whole or in part, may accept or reject, and shape action in practice accordingly.

*Too many dentists are presuming to be scientists,* and by their fumbling work mislead, create confusion, and mix up matters to a hurtful extent, attributable, possibly, to much guess-work and very little that may be called scientific.

More than a decade back it was proclaimed that through scientific investigations the cause of typhoid-fever had been discovered. The glad tidings were quickly heralded to all sections of country, and there was hope for freedom from the dread disease, but where is the physician who can say truthfully that typhoid fever is not as prevalent now and as well defined in every feature, and fatality as great, except in some hospitals, as before the cause microbe was discovered?

About the same period the distinguished Dr. Koch, of Germany, published to the world his discovery of the bacillus the cause of consumption, and created quite a general sensation and a decided craze with some physicians in many sections of country. There was a hurried rush of numberless physicians to Germany and back home again, with knowledge sought and a supply of the preventive and restorative lymph to be freely used as opportunity offered. Who of them after the lapse of ten years, with experience in the free use of the remedy, can mention a section of country or a defined locality where there has been a check of the disease or decrease of mortality through use of the saving lymph?

All scientific (so termed) discovery of causes and remedies for cure have not, when carefully investigated and thoroughly tested, proved as reliable and satisfactory as was desired and anticipated.

As in medicine and with physicians, so in dentistry and with dentists. The germ theory introduced and advocated by Dr. Miller, an able and distinguished scientist, and possibly more to be relied upon than any one who has written on the subject, has been accepted by a large majority of the most prominent men in the dental profession for some years past, and what of the results? Who can venture to say that caries of the teeth is not as common to-day as at the time Dr. Miller proclaimed his discovery and published his germ theory? So far there has been no evidence of check of caries, or abatement of its ravages. The disease prevails with destructive tendencies as before the notable discovery, and very likely will so continue indefinitely.

Within the past two years a prominent dentist, with some pretensions as a scientist, has proclaimed discovery of the cause (a specific bacillus) of pyorrhœa alveolaris. But, unfortunately for those afflicted with the disease and for dentists disposed to battle with causes to check progress and effect cure, the bacillus as cause (so represented) is not found, only deep down in pus-pockets when the disease is far advanced, with considerable destruction of the peridental membrane and alveolus and much discharge of pus, almost too far advanced for general success in treatment. Under such circumstances it is reasonable to predict that no practical benefits will be derived from the important or non-important discovery.

As with caries, after discovery of cause, pyorrhœa alveolaris will in all probability continue as before discovery of the specific bacillus, and for check of progress when well advanced (bacillus or no bacillus) nothing short of heroic treatment in the use of steel instruments will avail.

If these causes of caries and pyorrhœa alveolaris, as stated, are facts, reliably established upon a basis of correct scientific investigation on right lines for discovery of true cause, it will be possible and natural to anticipate as results, perceptible in a reasonable period of time, decided check of progress and cessation of extreme features of advanced stages of both. But let us wait and see "what we shall see."

In course of time scientific work on the line of discovery of causes by men of skilled attainments, as Dr. Miller and others, as scientists, there may be development of truths that will enable us to eradicate successfully cause of caries and other diseases, and in future years caries will be known only as a thing of the past; but I must say, in all candor, I think it very questionable.

Scientific work on lines of investigation for discovery and the revealing of hidden mysteries and facts is essential and the work must be encouraged and sustained, that truths pertaining to many subjects may be made known and fully comprehended for the general advancement of civilization and professions and trades of all types and grades. Without the aid of scientific work by scientists there can be no promise of advance to higher planes of progress. But in relation to dentistry in recent years there has been too much (scientific, so called) theorizing about discovery of causes and sundry features pertaining to practice, much that has not so far amounted to anything profitable in effect for check of disease and cure or improvement of practice, not even to the value of a penny.

Scientists, such as are popping up all over the country at present, are too numerous and incompetent, with rare exceptions, to be graded first class and accredited as reliable authority. There are but few of notable prominence, and some of them, like men in general in all callings, slip up occasionally and make laughable blunders, some so noticeable and conspicuous as to require prompt revision and correction. The most distinguished and reliable, with attainments unquestioned, are not beyond the reach of mishap and the possibility of varying from a true line of correct work and erroneous statements sometimes. Figures, microscopes, instruments, and chemicals will fail the most expert and skilful occasionally, consequently there are failures and misstatements that will lead astray if not corrected.

Hundreds of pages in dental journals and large volumes of accepted scientific works pertaining to dentistry and other subjects, and prized as valuable treasure in our bookcases, will in all probability, in less time than a decade or two, be discarded as unreliable and worthless, to make room for books on same subjects, more trustworthy—a natural consequence, as the result of progress in science.

So far the most of the scientific work of investigation pertaining to dentistry presented and accepted by the profession has been too immature to be accorded first rank or strictly reliable. However, the work of investigation will be continued, and every impetus that is given to it will the more surely secure to us gratifying results in fruit fully matured.

The revelation of facts, reliable and unchangeable, bearing upon dentistry and subjects pertaining to it is what we need and want, and will doubtless ultimately secure. Through scientific research and discoveries that may be made, it is possible that the causes of all the abnormal conditions of organs of the human body may be made known, and the various ailments that the human system is heir to may be successfully treated and checked in the stages of incipiency. Hence no prolonged chronic suffering, no extensive decay of teeth to be treated with large fillings or crowns. Then there will be need for neither physician nor dentist of the presentday status of qualifications and extravagant equipments for service as manipulators of the healing art.

Such advance and development of results as the ultimate outgrowth of scientific investigations may be anticipated as a reality, but in the may be distant future. But few of us, if any, now in practice can reasonably hope to witness the change involving the higher order of discoveries and professional skill in advanced practice, but we can and should willingly help the progressive work along, and pursue practice with the lights and aids we have, on best lines possible for advance and good results. We may feel assured that through the never-varying principles of evolution and the aid of scientific work, more matured, seals will be broken and mysteries revealed that will enable those following us to conduct practice on the more reasonable, humane and higher plane. All will then unite in harmonious acclaim with feeling of appreciation and gratitude, and generously proclaim all praise and honor is due and must be given to self-sacrificing scientists who have done so much, and so nobly in earnest, faithful work in the interest of humanity and the advancement of dentistry. This will be a pivotal point and the dawning of a new era in dental practice for preservation of teeth in a comparatively natural state.

The use of pluggers and forceps and other implements and features conspicuous in present practice will be discarded for the substitution of new features further advanced. The new and improved line of practice will be in accord with evolving science, regulated by a closer and more careful observance of the nature and requirements of the human system, its varied freaks and weaknesses, and requisite need for aid at all ages and under all circumstances. Such is reasonable to anticipate as possible, and must be worked for and hoped for.

We must anticipate and hope for the certainty of definite realization of advanced and highest features of attainment in the work of scientific investigation and superior skill and expert service in professional work, whether on lines of prevention or cure.

The zenith will be attained, time not definite, but, as a fact, ultimate, it is evident. What then will be the fate of some of the popular features in practice at the present time,—wholesale extraction of teeth, ornamental display fillings, gold caps, crowns and bridges, the use of numerous useless dentifrices, antiseptic and disinfectant mouth-washes, and the many worthless local anæsthetics? All of them must unquestionably be discarded for a line of practice more in keeping with the limited scope of the needs of the teeth and associate tissues, practice strictly in keeping with physical laws and for the best support of the human system from early childhood to advanced age. The result will be well-preserved teeth and gums and freedom from much physical discomfort, with a reasonable probability of increased longevity, a state of things desirable and quite as possible, and as reasonable to anticipate as many of the changes and advanced features in practice some of us have witnessed during the past half-century.

The change is needed and is pending. We may as well prepare for the inevitable.

